# Super-Resolution Imaging Approaches for Quantifying F-Actin in Immune Cells

**DOI:** 10.3389/fcell.2021.676066

**Published:** 2021-08-19

**Authors:** Evelyn Garlick, Steven G. Thomas, Dylan M. Owen

**Affiliations:** ^1^Institute of Cardiovascular Sciences, College of Medical and Dental Science, University of Birmingham, Birmingham, United Kingdom; ^2^Centre of Membrane Proteins and Receptors, University of Birmingham and University of Nottingham, Midlands, United Kingdom; ^3^Institute for Immunology and Immunotherapy, College of Medical and Dental Science and School of Mathematics, College of Engineering and Physical Science, University of Birmingham, Birmingham, United Kingdom

**Keywords:** actin, microscopy, super-resolution, fiber analysis, cytoskeleton

## Abstract

Immune cells comprise a diverse set of cells that undergo a complex array of biological processes that must be tightly regulated. A key component of cellular machinery that achieves this is the cytoskeleton. Therefore, imaging and quantitatively describing the architecture and dynamics of the cytoskeleton is an important research goal. Optical microscopy is well suited to this task. Here, we review the latest in the state-of-the-art methodology for labeling the cytoskeleton, fluorescence microscopy hardware suitable for such imaging and quantitative statistical analysis software applicable to describing cytoskeletal structures. We also highlight ongoing challenges and areas for future development.

## Introduction

The actin cytoskeleton is a key cellular component in many biological processes and is particularly implicated in a range of functions associated with immune cells. For example, actin polymerization is responsible for the spreading of lymphocytes over target antigen-presenting cells (APC), creating a large contact area (Dustin and Cooper, 2000), whereas cortical actin retrograde flow drives T cell signaling microclusters toward the synapse center through frictional coupling (Billadeau et al., 2007; DeMond et al., 2008; Hartman et al., 2009; Yu et al., 2013; Kumari et al., 2014; Ashdown et al., 2017). In cytotoxic lymphocytes, cortical actin at the immunological synapse must be remodeled to an actin mesh, which allows the release of cytotoxic granules (Carisey et al., 2018). Actin is also responsible for driving cell migration in response to cytokines (Samstag et al., 2003; Shannon et al., 2020) and processes such as phagocytosis (Castellano et al., 2001; May and Machesky, 2001).

For these reasons, there has been a long-standing goal to understand the architecture of the cytoskeleton in immune cells and numerous techniques have been deployed for this purpose. For example, transmission electron microscopy has been used to image the ultrastructure of cytoskeletal components (Svitkina, 2009). However, in recent years, optical microscopy has come to the forefront of these endeavors for several reasons. Firstly, optical microscopy is relatively non-invasive, allowing cytoskeletal architecture to be imaged in live cells and cytoskeletal dynamics to be captured and analyzed. Fluorescence microscopy is also specific, allowing particular proteins of interest to be labeled and observed (Lichtman and Conchello, 2005).

For many years, fluorescence microscopy has been held back by its intrinsic resolution limit of around 200 nm. The development of super-resolution (Hell and Wichmann, 1994; Betzig et al., 2006) approaches has broken this barrier and now allows cytoskeletal architecture to be imaged and quantified on the nanoscale. Combined with developments in probes, labeling technology, and in quantitative analysis, advances in microscope design are proving transformative to our understanding of the many diverse functions of the cytoskeleton in immune cell function.

Here, we will review the latest developments in cytoskeletal labeling, applicable microscope hardware, and the latest quantitative analysis methodology.

## Labeling

For fixed samples, the gold standard label for filamentous (F-) actin is phalloidin. A bicyclic heptapeptide initially isolated from the death cap mushroom (*Amanita phalloides*), phalloidin is an F-actin stabilizing toxin first used to visualize F-actin in 1979 (Wulf et al., 1979). Fluorescent phalloidins are widely used to visualize F-actin in many applications. Interestingly, the specific fluorophore conjugated to phalloidin can affect imaging outcomes. For example, phalloidin-AlexaFluor 488 has been shown to provide superior detail and longevity of stain over other derivatives like AlexaFluor 405 (DesMarais et al., 2019). However, the choice of label will, to some degree, be application dependent.

Since phalloidin cannot permeate live cells, fixation is important. Paraformaldehyde (PFA) can lead to disruption of the actin cytoskeleton (Leyton-Puig et al., 2016; Pereira et al., 2019) so use of a cytoskeletal stabilizing buffer is recommended. This results in more faithful preservation of actin architecture. Methanol fixation, while often favored for microtubule preservation, results in significant actin disruption and should be avoided (Whelan and Bell, 2015; DesMarais et al., 2019).

There are also numerous staining options for live imaging of actin. For a thorough review of actin labeling, see Melak et al. (2017). Direct tagging of actin subunits with recombinant fluorescent proteins (FP) is possible and has been used extensively. These constructs must be expressed exogenously, and although capable of co-polymerizing with the untagged endogenous actin (Westphal et al., 1997), FP-actin kinetics and behavior can be impeded (Aizawa et al., 1997; Deibler et al., 2011; Nagasaki et al., 2017) and non-integrated monomers can generate background. Tagging actin binding proteins (ABPs) is therefore a more attractive option. Their capacity to bind more efficiently to actin filaments than monomers results in better identification of the filamentous cytoskeleton. Examples of ABPs include derivatives of the calponin homology domain of utrophin (Burkel et al., 2007), a short peptide from the rat inositol 1,4,5-trisphosphate 3-kinase (ITPK) called F-Tractin (Schell et al., 2001) and a 17-amino acid peptide derived from Abp140 called Lifeact (Riedl et al., 2010). The generation of a GFP-Lifeact transgenic mouse line (Riedl et al., 2010) has allowed live actin imaging *in vivo*, along with tissue-specific expression using a cre inducible mouse line (Schachtner et al., 2012). Additionally, Lifeact can be adapted for use with single-molecule imaging strategies such as IRIS (Kiuchi et al., 2015). While widely used, actin binding protein derivatives should still be approached with caution. For example, Utrophin derivatives can induce actin aggregates in both the nucleus and cytoplasm, and recent findings suggest that Lifeact-TagGFP2 can disrupt actin architecture in a dose-dependent manner (Flores et al., 2019).

SiR actin is an F-actin probe based on jasplakinolide, an actin binder and potent promoter of nucleation. The silicon rhodamine (SiR) probe is fluorogenic (Lukinavičius et al., 2014), cell permeable, and can be added to live cells in culture without washing. As SiR actin is a derivative of jasplakinolide, however, higher concentrations can impact dynamics significantly (Lukinavičius et al., 2014).

Newer labeling strategies like nanobodies and affimers have generated interest in recent years. For example, fluorophore fused nanobodies (“chromobodies”) can retain labeling functionality when expressed intracellularly, allowing for specific live-cell imaging (Rothbauer et al., 2006; Schiavon et al., 2020). In general, nanobodies offer significant antigen specificity and reduced size over standard antibodies. Affimers are synthetic reagents generated from non-antibody scaffold proteins such as Adhirons (Tiede et al., 2014). Lopata et al. (2018) demonstrated the identification and validation of four affimers that specifically recognize F-actin. Their versatility and potential for live-cell application make them important reagents for the future (Lopata et al., 2018). Some of the more widely used labeling strategies are summarized in Table 1.

**TABLE 1:** Summary of the advantages and disadvantages of different actin labeling strategies.

	Label	Uses	Pros	Cons
Phalloidin	F-actin binding peptide (Wulf et al., 1979)	Fixed F-actin labeling	Gold standard for fixed actin imaging	Unsuitable for studying actin dynamics in live cells
Tagged β-actin	Exogenously expressed fluorescently tagged actin subunits (Westphal et al., 1997)	Live imaging	Can incorporate into endogenous filaments	Can affect dynamics G-actin also visible
Utrophin and F-tractin	FP-tagged region of actin binding proteins (Schell et al., 2001; Burkel et al., 2007)	Live imaging	Does not require direct tagging of actin Does not require over-expression of actin monomers Does not label G actin	Can affect dynamics
LifeAct	17 amino acid peptide derived from Abp140 (Riedl et al., 2008)	Live imaging	Does not require direct tagging of actin Does not require overexpression of actin monomers Small probe adaptable for many techniques	Can affect dynamics G-actin can be labeled
SiR Actin	Silicon Rhodamine probe conjugated to a jasplakinolide derivative (Lukinavičius et al., 2014)	Live imaging	Fluorogenic Cell permeable Concentration is titratable	High concentrations can affect dynamics
Chromabodies	Fluorophore fused anti-actin nanobodies (Rothbauer et al., 2006)	Live imaging	Significantly improved specificity over antibodies Reduced size over antibodies	G-actin can be labeled
Affimers	Synthetic reagents generated from non-antibody scaffold proteins (Lopata et al., 2018)	Live and fixed imaging	Small probe size Potential for structural sub- selectivity	Not yet used routinely Further screening required

## Microscopy Techniques

Simple assessment of gross actin structures is easily achieved using diffraction-limited imaging systems. Abbe’s law, often referred to as the diffraction limit, describes how the minimum resolution achievable in a system is dependent on the numerical aperture of the lens and the wavelength of the incident light. Due to these limitations, the maximum resolving power of diffraction-limited systems is ∼200 nm laterally and ∼600 nm axially. One of the simplest fluorescence techniques, wide-field epifluorescence imaging, involves illumination of the full sample. This results in the camera or detector receiving all emitted light, both in and out of focus, which can reduce the images’ signal-to-noise ratio (SNR) significantly as well as limit optical sectioning capacity. Epifluorescence microscopes are, however, simple systems capable of high-speed acquisition, and combination with image post-processing like deconvolution can provide powerful insight into dynamic actin processes.

Total internal reflection fluorescence microscopy (TIRF) is achieved by angling the incident light such that it is entirely reflected, rather than refracted. At this so-called critical angle, an evanescent wave is generated that decays exponentially with depth, restricting fluorophore excitation to approximately 200 nm from the coverslip and reducing the out-of-focus background. TIRF is therefore often the technique of choice when imaging dynamic actin in adherent cells.

The dynamics of actin in T cell synapses are well imaged with TIRF. For example, Ritter et al. (2017) used TIRF imaging to follow cortical actin recovery in cytotoxic T cell synapses following lytic granule secretion (Ritter et al., 2017). Time lapse imaging allowed assessment of the cortical actin structure pre- and post-secretion, providing insight into the tightly regulated process. The relatively lengthy time courses (<10 min) with high temporal resolution (2.4-s intervals for two color experiments) were possible due to TIRF microscopy. Variation in the actin structure of stable and unstable immune synapses has also been investigated using TIRF, including on supported lipid bilayers (Kumari et al., 2020). Wagh et al. (2020) used TIRF to quantify actin reorganization in the immune synapse of CD8 effector T cells relative to Bcl10 expression. Analysis resulted in the identification of two phases of dynamics—a faster initial stage and slower latter, both of which were significantly faster in Bcl10^–/–^ cells. In this case, the high temporal resolution possible with TIRF was essential.

Confocal microscopy is another workhorse of diffraction-limited imaging. By applying a pinhole in front of the detector, out-of-focus light is discarded. Point scanning microscopes must raster scan across the sample, meaning the method is less suited to fast imaging. Laser intensity is often increased too. Spinning disk confocal microscopes can overcome some of the limitations on speed and phototoxicity by inclusion of an array of microlenses, allowing multiple focused beams of light to be swept across the sample to reduce intense laser exposure. Resonant scanning confocal microscopy also improves acquisition speed in single point scanning systems, using a resonant mirror scanner that oscillates at a fixed frequency to improve framerate to video rate (30 fps) and beyond. Confocal microscopes are a solid choice for actin imaging given their advantages over widefield microscopy and common presence in core facilities.

Lightsheet microscopy is regarded as one of the gentlest techniques for live-cell imaging. A sheet of light, hundreds of nanometers thick, is generated perpendicular to the imaging objective and moved through the sample. This planar illumination significantly reduces out-of-focus light, improving signal to noise and reducing phototoxicity. This technique also permits rapid capture of 3D data. A variant of this concept, lattice light sheet (Chen et al., 2014) microscopy, replaces the conventional sheet with an optical lattice, further reducing phototoxicity and improving imaging speed. Examples of light-sheet imaging of the cytoskeleton in immune cells include imaging the retrograde flow of cortical actin in cell–cell synapses (Ritter et al., 2015) and actin protrusions in cytotoxic T cells (Tamzalit et al., 2019) and neutrophils (Fritz-Laylin et al., 2017).

**FIGURE 1:**
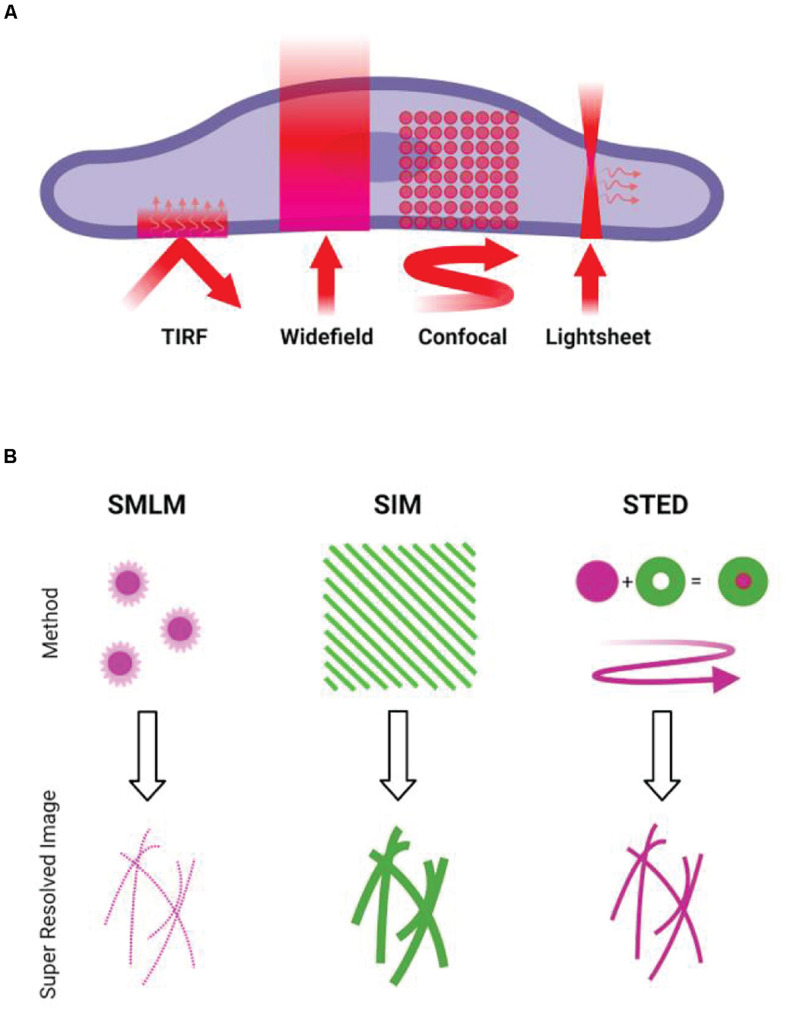
Overview of microscopy techniques commonly used for imaging actin. **(A)** Diffraction-limited techniques. TIRF, showing the incident light and resulting evanescent wave. Widefield epifluorescence, showing illumination of the full volume of the specimen. Confocal, showing the rastering of the scanning focal point. Lightsheet, showing the sheet illumination and resulting detection at an angle to illumination. **(B)** Illustrations of the concepts behind common super-resolution techniques. SMLM requires the stochastic activation and localization of single fluorophores, building up a pointillist image of the structure. SIM involves projection of patterned illumination at multiple angles, using the resulting Moiré fringes to reconstruct a higher frequency information. STED uses a depletion laser that surrounds the point of illumination, reducing the size of the point spread function of the illumination beam.

While the diffraction-limited techniques are widely used to study immune cells, in many cases, cytoskeletal structures need to be studied on the nanoscale. There are now multiple techniques capable of breaking the diffraction limit (Illustrated in Figure 1). Fluorescence microscopy can obtain resolutions only previously possible with electron microscopy, with single-molecule localization microscopy (SMLM) techniques routinely reporting resolving power of up to 10 nm.

Structured illumination microscopy (SIM) allows extraction of higher-resolution information by illuminating the sample in a known pattern—usually stripes, but variations like lattice SIM use differing illumination patterns. The pattern is translated and rotated across several frames, and the interference of this light pattern with the sample generates so-called Moiré fringes. High-frequency information is then extracted during computational reconstruction, generating images with roughly a 2× increase in resolution. Reconstruction artifacts can be an issue, especially in thicker or more densely labeled samples [see Demmerle et al. (2017), for images and explanations of common issues]. Critical inspection of reconstruction results, as well as using packages such as SIMCheck (Ball et al., 2015), is best practice. Structured Illumination can also be applied to lattice light sheet microscopy, allowing rapid and gentle acquisition of live-cell super-resolved data (Wagh et al., 2020). In immune cells, SIM has been used with TIRF illumination to image cortical actin labeled with phalloidin (Ritter et al., 2017) and in live cells using LifeAct (Ashdown et al., 2017), both at the T-cell immunological synapse. Colin-York used TIRF SIM to show differences between cytoskeletal behavior in primary and immortalized T cells (Colin-York et al., 2020) and Brown used SIM to analyze cortical actin at the sites of lytic granule release at NK cell synapses (Brown et al., 2011).

Another point scanning technique, stimulated emission depletion (STED) microscopy (Hell and Wichmann, 1994), achieves super-resolution by selectively depleting fluorophores around a central focal point. As the size of the excitation point is reduced by a doughnut-shaped depletion laser, resolutions of 30–80 nm can be achieved. Optical sectioning is also a particular strength, as the technique is point scanning, and diffractive optical elements can be introduced to generate an additional depletion doughnut in *z* (Klar et al., 2000). Intense laser power in the depletion beam is required, making phototoxicity a key consideration. Recent developments in this technique (MINFLUX) (Balzarotti et al., 2017) reduces the phototoxicity caused by the high laser intensities of STED. Fritzche used STED to image the actin cytoskeleton in immune cells (Fritzsche et al., 2017) and Clausen used the method to analyze the relationship between the actin cortex and the membrane (Clausen et al., 2017). Carisey showed the applicability of STED (as well as TIRF SIM) to image the dynamics of actin in NK cells (Carisey et al., 2018) and Rak used similar methodology to analyze the architecture of the actin cortex during lytic granule release (Rak et al., 2011).

Single-molecule localization microscopy techniques boast impressive resolutions. All techniques under this umbrella rely on detection and mathematical localization of individual fluorophores, but achieve this stochastic imaging in different ways. Key examples include stochastic optical reconstruction microscopy (STORM) (Rust et al., 2006), which utilizes the photophysical properties of certain fluorophores (most often Alexa-Fluor 647) in order to push them in and out of a transient dark state. Photoactivated localization microscopy (PALM) (Betzig et al., 2006) relies on photoswitchable proteins to illuminate limited subsets of the sample. Points accumulation for imaging in nanoscale topography (PAINT) (Sharonov and Hochstrasser, 2006) makes use of repeated association and dissociation of the labeled probe, be that through transient interaction between complementary DNA oligonucleotides (DNA-PAINT) (Schnitzbauer et al., 2017), the most commonly implemented option, and IRIS uses the repeated binding of the LifeAct peptide (Kiuchi et al., 2015). IRIS was later used with quantitative analysis to dissect the architecture of cortical actin at the T-cell immune synapse (Peters et al., 2018). Actin has been successfully imaged using all of these techniques. Given the small size of individual actin filaments (∼7 nm), the approximate resolution of 100 nm of techniques like SIM can still obscure significant detail in the actin architecture, which can be revealed with SMLM. As yet, however, this high resolution comes at the expense of live-cell imaging. Most examples are of fixed cells, as toxic blinking buffers (mostly for dSTORM), high laser intensities, and long acquisition times needed limit live applicability.

Many live super-resolution techniques require specialized optical setups and can have limitations in terms of labels and imaging speed. Development of algorithmic approaches to live super-resolution microscopy allows circumvention of some of these issues. Examples include SOFI (super-resolution optical fluctuation imaging, which analyzes temporal fluctuations of the emitters) (Dertinger et al., 2009) and super-resolution radial fluctuations (SRRF) (Gustafsson et al., 2016). SRRF, which uses fluorescence fluctuations over time to calculate radiality on a subpixel basis, is a widely applicable technique, being capable of taking raw widefield, TIRF, confocal, and even SMLM data. The technique has been demonstrated as a way to image LifeAct-GFP labeled actin in T-cell immunological synapses (Gustafsson et al., 2016), with significant improvement in resolution.

Correlative approaches, where multiple imaging techniques are applied to the same cell, are becoming more prevalent in the field. Correlative light and electron microscopy (CLEM) provides the as-yet unparalleled resolving power of electron microscopy techniques while also overcoming some of the labeling limitations inherent in using EM alone. Much of this work has been done outside of immune cells, for example, in neurons, elucidating new nanoscale cytoskeletal structures (Vassilopoulos et al., 2019); however, super-resolution CLEM has been used to image multiple proteins relative to the actin structure around clusters of podosomes in dendritic cells (Joosten et al., 2018), providing insight into differential association of particular proteins with different structures in the podosome. Correlative approaches do extend beyond EM, however. For example, a recent study used SMLM in conjunction with AFM to image podosomes in THP-1 cells (Hirvonen et al., 2020), opening possibilities for dual structural and mechanobiological mapping. The main recent imaging strategies used to image F-actin are summarized in Table 2.

**TABLE 2:** Summary of key microscope techniques applied to cytoskeletal imaging.

Imaging method	Advantages	Disadvantages	Immune cell use examples
TIRF	Improved SNR Rapid imaging	Diffraction limited Focal plane limited to ∼200 nm proximal to coverslip	Ritter et al., 2017; Wagh et al., 2020
Light Sheet	Good optical sectioning Improved SNR Rapid and gentle imaging	Diffraction limited if not SIM-lattice) Specialized system required	Ritter et al., 2015; Fritz-Laylin et al., 2017; Tamzalit et al., 2019
SIM	2× improvement in resolution vs. diffraction limited, axially and laterally (3D SIM)	Potential for reconstruction artifacts Acquisition speed can be limiting for live imaging Specialized system required	Brown et al., 2011; Ashdown et al., 2017; Colin-York et al., 2020
STED	30–80 nm resolution No computationally intensive post-processing	High laser powers necessary for depletion beam Specialized system required	Rak et al., 2011; Clausen et al., 2017; Fritzsche et al., 2017; Carisey et al., 2018
SMLM	20 nm + resolutions, up to ∼10 nm localization precision Data obtained as a point cloud	Lengthy image acquisitions can be necessary Sample preparation can be complex Potential for reconstruction artifacts	Kiuchi et al., 2015; Hirvonen et al., 2020
Computational techniques (e.g., SRRF, SOFI)	Simple image acquisitions No specialized hardware necessary	Potential for reconstruction artifacts	Gustafsson et al., 2016

## Quantification

Once sample preparation, labeling, and imaging have been performed, images must be analyzed to extract biologically meaningful, quantitative data. The type of analysis performed depends largely on the biological question being asked. For example, a key differentiator is whether dynamic, live-cell data are being analyzed. Dynamic imaging analysis may involve quantifying flow or the stability of a particular architecture (Ashdown and Owen, 2018; Shannon et al., 2020), for example. In static structures, far richer descriptions of the actin architecture are generally possible.

**FIGURE 2:**
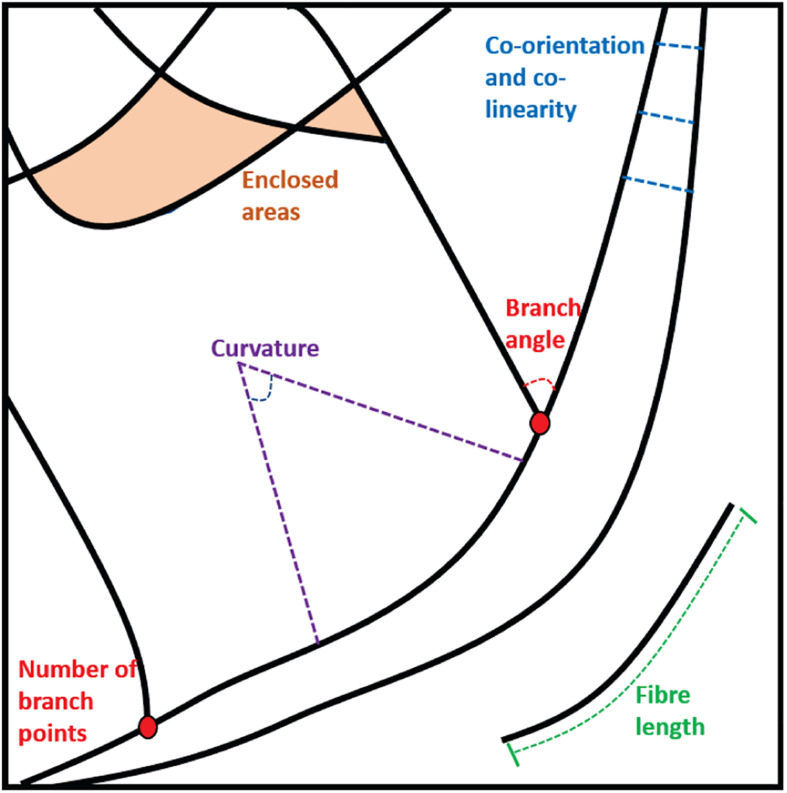
Examples of the parameters that can be extracted from static fiber analysis: Enclosed areas, branch points and angles, fiber lengths and measures of curvature, co-orientation, and co-linearity.

For components of the cytoskeleton that are typically fibrous structures, the first step is usually to attempt to trace the fibers themselves to generate a mathematical representation of the architecture. This can then be interrogated to extract specific descriptive features. Examples of descriptors that might be desirable when quantifying a fibrous pattern include the number or density of fibers in a given area or volume, the lengths of fibers, or measures of co-orientation and co-linearity between fibers. They may also include the sizes and shapes of enclosed areas between fibers and analysis of branching angles and cross-points as well as curvature measures. These different parameters are illustrated in Figure 2.

There are many competing algorithms capable of analyzing fibrous architecture and providing these descriptions, but they can generally be divided into two groups—those that work on pixelated images such as those derived from widefield, TIRF, confocal, STED, or reconstructed SIM images, and those that work with pointillist data—the *x*,*y* coordinates derived from SMLM imaging.

For methods that produce pixelated images, methods for analyzing actin distributions have existed for some time. Many of the algorithms are related to those that can be used to trace other fibrous structures—dendrites or axons from neuronal images are another common example (Kayasandik et al., 2018). Many are available in image analysis packages such as ImageJ (Schneider et al., 2012) and more exist in various proprietary packages (Schindelin et al., 2012). They often take the form of a ridge estimating algorithm. Many of these, however, begin with some form of cleanup of the raw image—for example, noise filtering. Recently, machine-learning approaches have proven to be well suited for this task and user-friendly packages capable of using machine learning to clean and enhance cytoskeletal images have become available (von Chamier et al., 2021).

One particularly widely applied method of analyzing actin fibrous architecture from pixelated images in immune cells is to quantify the size distributions of clearances between fibers. For cortical actin, this parameter represents a measure of what size of object could traverse the meshwork when traveling from an intracellular pool to the plasma membrane. Such a method has been used to analyze clearances in NK cells (Rak et al., 2011), showing that cortical actin must be cleared for lytic granule release, and the application of such methodology to live-cell imaging of the cytoskeleton allowed the analysis of the dynamics of this clearance process (Carisey et al., 2018).

Zhang et al. (2017) described a method for extracting quantitative descriptions of microtubule architecture from super-resolution SMLM data, provided those data are first converted into pixelated images. Here, filamentous features are enhanced using filters and biologically derived constraints are used to join filament sections together. This allowed the extraction of many of the desirable descriptors: filament length, number of filaments, and so on (Zhang et al., 2017). Somewhat similarly, Kittisopikul also use filters to detect fibrous structures; however, the use of a tunable filter allows particularly detailed analysis of fiber junctions (Kittisopikul et al., 2020). Very recently, one of the most complete algorithms for analyzing fibrous patterns from pixelated images has been developed. FiNTA (Flormann et al., 2021) is based on the vector tracing of grayscale images. It provides rich descriptors of fibrous patterns such as connectivity between fibers, hold sizes and shapes, filament density, and length and measures of curvature.

One way of describing fibrous architecture directly from SMLM-derived point coordinates is to re-purpose statistical methodology designed to analyze other types of structure such as molecular clusters. One such method is Ripley’s K-function, which is based on counting the number of points within ever-increasing circles, centered on each data point. Peters et al. (2018) showed that when a fibrous cytoskeletal network is regular and periodic, this periodicity becomes evident in the Ripley’s K-function curves (Peters et al., 2017). The K-function can therefore be used to measure how regular or chaotic a fibrous pattern is (Peters et al., 2017). Moreover, for immune cell synapses, which are generally radially symmetric, especially in artificial cases such as on a hard surface or a planar-supported lipid bilayer, the method could describe the proportion of tangential and radial fiber orientations.

To extract richer descriptors, algorithms have been developed that trace fiber positions through point clouds (Peters et al., 2018). Such algorithms start at a particular point on a fiber and then attempt to “walk” along the structure, analogous to attempting to navigate along a mountain ridge. This allowed the extraction of fiber lengths, branch points, and so on. Other methods, for example, cluster analysis by Voronoi Tessellation, are applicable to fibers (Levet et al., 2015) as are machine-learning approaches (Williamson et al., 2020).

## Conclusion

The actin cytoskeleton is a diverse structure. Its organization and dynamics have a profound effect on the regulation of immune cells. Here, we have reviewed optical imaging approaches for this purpose, examining labeling strategies, imaging hardware, and the associated statistical analysis. Taken together, these allow the structure and dynamics of the cytoskeleton to be quantitatively described, even on scales far below the resolution of conventional optical imaging.

Despite the recent progress, several aspects of the role of the cytoskeleton in immune cells remain to be uncovered and further developments in imaging techniques might shed light on these questions. One interesting aspect concerns the role of cortical actin in regulating the distributions and dynamics of membrane proteins. For example, it is known that many proximal signaling proteins on the surface of NK cells, B cells, and T cells are clustered on the nanoscale and show various kinds of constrained diffusion, especially at immunological synapses (Fooksman et al., 2010; Yokosuka and Saito, 2010; Pageon et al., 2013). Frameworks such as the picket fence model (Fujiwara et al., 2016) seek to explain the role of cortical actin in these processes. However, even for super-resolution techniques, the density of fibers in the cortex can be a challenge to accurately describe, especially given their dynamic nature. Correlative methods, ultra-high-resolution imaging, and live-cell SMLM could be applicable to this problem (Henriques et al., 2011; Balzarotti et al., 2017; Griffié et al., 2018; Cnossen et al., 2020).

Another aspect frequently left understudied is the 3D organization of the cytoskeleton. In immune cell processes such as phagocytosis, actin is necessarily remodeled in 3D. 3D SMLM and fiber analysis have been attempted (Peters et al., 2019), but advancements in other 3D modalities, including light sheet, would also be advantageous (Sapoznik et al., 2020).

Finally, a perhaps undervalued area is the mathematical modeling of the resulting data. A common goal is often not to image or understand the actin organization in any particular cell, but to be able to model and predict the organization in an average cell or a population of cells. To do this, statistical modeling approaches and machine learning require large amounts of data, and so high-throughput and high-content imaging systems, centered on automation, will be key.

## Author Contributions

All authors listed have made a substantial, direct and intellectual contribution to the work, and approved it for publication.

## Conflict of Interest

The authors declare that the research was conducted in the absence of any commercial or financial relationships that could be construed as a potential conflict of interest.

## Publisher’s Note

All claims expressed in this article are solely those of the authors and do not necessarily represent those of their affiliated organizations, or those of the publisher, the editors and the reviewers. Any product that may be evaluated in this article, or claim that may be made by its manufacturer, is not guaranteed or endorsed by the publisher.
